# A novel method for the translation and cross-cultural adaptation of health-related quality of life patient-reported outcome measurements

**DOI:** 10.1186/s12955-023-02089-y

**Published:** 2023-01-31

**Authors:** Catherine J. P. Teig, Malcolm J. Bond, Margreth Grotle, Møyfrid Kjøllesdal, Susan Saga, Milada S. Cvancarova, Marie A. Ellström Engh, Angelita Martini

**Affiliations:** 1 The Pelvic Floor Centre, Division of Surgery, Akershus University Hospital, Lørenskog, Norway; 2 School of Medicine, Flinders University, Adelaide, Australia; 3 Faculty of Health Science, OsloMet – Oslo Metropolitan University, Oslo, Norway; 4 Research and Communication Unit for Musculoskeletal Health, Oslo University Hospital, Oslo, Norway; 5 Drammens Gynaecology Private Clinic, Drammen, Norway; 6 Department of Public Health and Nursing, Norwegian University of Science and Technology, Trondheim, Norway; 7 Department of Biostatistics, University of Oslo, Oslo, Norway; 8 Department of Obstetrics and Gynaecology, Akershus University Hospital, Lørenskog, Norway; 9 School of Population and Global Health, University of Western Australia, Perth, Australia

**Keywords:** Delphi method, Expert panel, Translation, Cross-cultural adaptation, Pelvic floor dysfunction, PFDI-20, PFIQ-7

## Abstract

**Background:**

This paper presents a novel methodology for translation and cross-cultural adaptation of health-related quality-of-life patient-reported outcome measures, incorporating the Delphi method. Specifically, we describe the process of translating the Pelvic Floor Distress Inventory-20 and Pelvic Floor Impact Questionnaire-7 from English to Norwegian using this method.

**Methods:**

The multistep translation method combined the European Organization for Research and Treatment of Cancer Quality of Life guidelines, an Expert Panel review, and the Delphi method. It comprised two independent forward- and back-translations. While the bilingual pelvic floor Expert Panel ensured rigorous cross-checking and effective cross-cultural adaptation, the addition of the Delphi method (comprising the attributes of anonymity, controlled feedback, and statistical group response) further established consensus on translated items.

**Outcomes:**

The application of the Delphi method in the Expert Panel phase proved adequate in producing comprehensible intermediate Norwegian versions ready for pilot testing. The Expert Panel reviewed the comments made by patients completing the instruments and offered advice to allow final translated versions to be produced and tested for measurement properties. This iterative approach, internal logic, and anonymity between rounds improved the evaluations that the panel members provided, which in turn enhanced the final translated Patient Reported Outcome Measures (PROMs).

**Conclusions:**

To our knowledge, this work represents the first demonstration of the application of an Expert Panel review incorporating a Delphi method to assess health-related quality-of-life instruments. The controlled feedback approach, iterative nature, internal logic, and anonymity of the Delphi consensus method appeared to ensure a good cross-cultural adaptation of these PROMs.

## Highlights

- Efforts to ensure a good translation and cross-cultural adaptation of the patient-reported outcome measurement PFDI-20 and PFIQ-7 from English to Norwegian led to the development of a new methodology using a Delphi method with an expert panel.
- To the author's knowledge, this is the first study to use this specific methodology. The rigorous documentation process, controlled feedback approach, iterative nature, and internal logic of the Delphi consensus method appeared to contribute to improved translation results, ensuring good cross-cultural adaptation of these instruments. A simple translation or poor translation of a HRQOL PROM may yield misleading information or fail to identify important clinical changes.

## Introduction

Patient-reported outcome measurements (PROMs), including those that assess health-related (HR) quality of life (QoL), are commonly developed within a single country-specific context, logically carrying with them embedded linguistic and cultural nuances. The goal of effective translation and cross-cultural adaptation is therefore to acknowledge these features to provide a reliable and valid alternative for the target language and/or culture, thus ensuring equivalence between the source and the target versions of HRQoL PROMs. The result should increase the chance that the data will be accurate, for example, in identifying an important clinical change [1].

Ensuring equivalence between the source and target versions of instruments begins with the choice of an appropriate translation methodology [1]. Although a range of translation methods have been documented, including back-translation [2], and cognitive interviewing (pilot testing) [2], no consensus has established a gold standard [2]. Nevertheless, the preference for multistep rather than single-step methods is clear and recommended by both the International Society for Pharmacoeconomics and Outcomes Research and the European Regulatory Issues on Quality of Life Assessment Group [2].

In most multistep translation procedures, a key component is a multidisciplinary committee review (termed an “Expert Panel” in our methodology) [3]. Although methodologies vary in how they incorporate experts, face-to-face group meetings with the researchers are common [4]. These meetings are beneficial for recording opinions concerning equivalence and resolving items perceived as discrepant. However, face-to-face dialogue can be a disadvantage when a dominant personality or personalities are present or if inappropriate group pressure toward conformity becomes evident [5, 6].

One subtle variation on the Expert Panel is the Delphi method which incorporates the concepts of anonymity, controlled feedback, and statistical group response [6]. Anonymity is potentially advantageous in avoiding the influence of dominant personalities and group pressure for conformity. Several studies have employed the Delphi method, relying on interviews in conjunction with an Expert Panel in the development of health-related instruments [7, 8]. However, we are aware of no studies that have used a combined Expert Panel and Delphi method in the translation and linguistic validation of instruments.

### The study context

Condition-specific HRQoL PROMs are becoming increasingly useful tools for identifying and assessing patient symptoms and QoL [9, 10]. In the Norwegian language, there are currently few measures available for assessing pelvic organ prolapse (POP) and pelvic floor dysfunction. In this group of conditions, pelvic organ prolapse usually coexists with other pelvic floor dysfunction symptoms (e.g., lower urinary tract and bowel) [9].

The options were to develop new instruments or adapt existing instruments validated in another language [3]. If feasible, the latter is preferable because it provides a basis for a cross-cultural comparison of data. The translations allow Norwegian-speaking clinicians to assess their performance [11] and treatment of patients against international benchmarks.

Two common PROMs available in English are the 20-item Pelvic Floor Distress Inventory (PFDI-20) and 7-item Pelvic Floor Impact Questionnaire (PFIQ-7) [12]. Both have moderate to excellent reliability, validity, and responsiveness to change both generally and when tested against their respective longer versions [12]. Given these observations and their applicability in both clinical and research settings, the PFDI-20 and PFIQ-7 are ideal condition-specific HRQoL measures for assessing POP and pelvic floor dysfunction in Norwegian samples [12].

### Summary

The method and outcomes to be reported involved the translation, assessment of equivalence of the PFDI-20 and PFIQ-7. This involved a novel multistep translation method, which combined the European Organization for Research and Treatment of Cancer (EORTC) translation guidelines [13], an Expert Panel review [3], and the Delphi method [6]. The translation process was based on the EORTC translation guidelines (two independent forward translations, reconciliation, and two back-translations). While the bilingual pelvic floor Expert Panel ensured rigorous cross-checking and effective translation and cultural equivalence, the addition of the Delphi method (comprising the attributes of anonymity, controlled feedback, and statistical group response) further established consensus on translated items and moderate the interaction between the expert panelists. We extended the latter approach (i.e., Delphi Method) by adding a physical meeting with the goal of achieving consensus on items of discrepancy.

## Method

Translation and cross-cultural adaptation of the Norwegian PFDI-20 and PFIQ-7 consisted of seven steps: forward translations into Norwegian, synthesis of translations, back-translations, back-translation review, expert panel using the Delphi Method, and pilot test of Intermediate Version 2.0 with a sample of 20 patients with symptomatic pelvic organ prolapse (POP). After pilot testing, it was sent to the Expert Panel which reviewed comments from the patients, rendering the final translation Version 3.0. The testing of the psychometric properties of Intermediate Version 3.0 was conducted using a sample of 205 women with POP [14]. Test and re-test reliability, internal consistency, content validity, construct validity using hypotheses testing, ceiling and floor effects, responsiveness, and interpretability were all evaluated.

### Translation and cross-cultural adaptation process

**Fig. 1:**
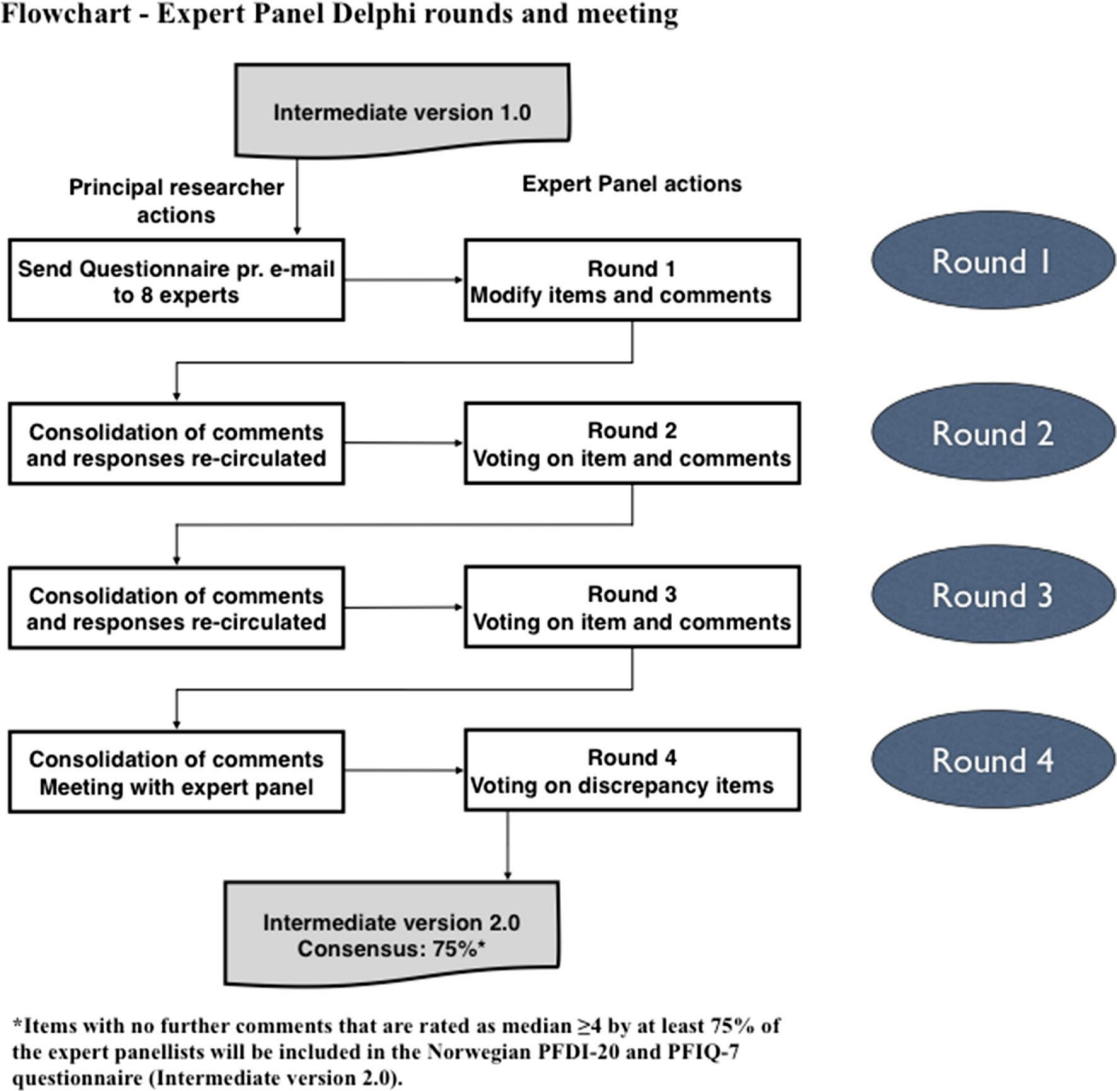
Sequence of events during the four rounds

The European Organisation for Research and Treatment of Cancer (EORTC) Quality of Life Group (QLG) comprises international researchers who focus on the development and translation of questionnaires within cancer practice and research. To ensure appropriate translation and cultural equivalence of a measure two forward translations, a reconciliation phase, two backward-translations, and pilot testing are required [15]. Specifically, the methodology for this study was based on the 2009 guidelines [13] with the addition of an Expert Panel review [3] using a Delphi method. Figure 1 presents a schematic representation of the Expert Panel Delphi rounds and a final face-to-face meeting.”

### Ethics approval

Ethics approval for this methodology and the collection of quantitative data for testing the reliability and validity of the resultant Norwegian instruments [14] was granted.

### Initial translation

Following permission from the authors of the original PFDI-20 and PFIQ-7, which also involved an inquiry about any known translation difficulties, two native speaking Norwegian professional translators (from a translation agency), with high English fluency, who were familiar with medical terminology and patient language, conducted the forward translation independently. The translation coordinator compared the resulting translations, which were reconciled by resolving any items of discrepancy with the forward translators to achieve equivalence [13]. The translation coordinator was a bilingual health researcher with expertise in both pelvic floor dysfunction and translation methodology.

For further quality control, a single translated version was then agreed upon and back-translated by two English speaking Norwegian professional translators with high Norwegian fluency who were familiar with medical terminology and patient language independently conducted the back- translation. The translation coordinator then compared the resulting translations, which were reconciled by resolving any items of discrepancy with the translators [13]. This step further verified the equivalence between the English and Norwegian versions. Following consensus (between the translation coordinator and translators) that the back-translated instruments were equivalent, Norwegian Version 1.0 of the PFDI-20 and PFIQ-7 were considered ready for the Expert Panel review. The expert panel review is comprised of two phases: an expert panel review using a Delphi method of the intermediate version 1.0, followed by the expert panel review of the intermediate version 2.0.

### Expert panel

The Expert Panel comprised gynecologists, colorectal surgeons, a urologist, a physiotherapist, and a nurse [7, 16]. All members had a relevant background and expertise in pelvic floor dysfunction [3, 16]. They were bilingual, with expertise in pelvic floor dysfunctions [3], and were working in a multidisciplinary hospital-based pelvic floor center. Based on recommendations by Dalkey and Thangaratinam [6, 17], eight people were invited to join the Expert Panel.

Version 1.0 of the translated scales was sent, by mail or electronically, to each member of the Expert Panel who responded either by email and/or telephone. The role of the panel was to verify semantic, idiomatic, experiential, and conceptual equivalence between the source and Norwegian versions of the PFDI-20 and PFIQ-7 [3, 14]. Members also were asked to assess comprehensibility, readability, and specific domain terminology, identify discrepancies within any items, and modify or reject items. The feedback obtained (during Rounds 1–4) allowed for production of a cross-culturally appropriate Version 2.0 for pilot testing.

There were no drop-outs in this study. Measures to reduce the dropout rate among the 8 panelists included using the expert panel`s preferred form of communication (i.e., e-mail or telephone) and continually working around the panelists busy schedule.

### Delphi method

As illustrated in Fig. 1, the Delphi method allowed for several rounds (rounds 1–3) and if required, a final meeting of the Expert Panel. The aim was to establish the extent of agreement across the panel and reach consensus if possible. After Round 3, if substantial disagreement remained on any items, the Round 4 action became a face-to-face meeting [7]. Further, as noted above, the Delphi method embodies the attributes of anonymity, controlled feedback, and statistical group response [6]. For anonymity, the panelists did not know which panelists had provided which responses. This anonymity was maintained through independent communication between panel members and the translation co-ordinator. Controlled feedback involved the use of iterative feedback, and summaries of results from previous rounds were communicated to all Expert Panel members.

Statistical group response pertains to the use of a quantitative measure of panel members’ opinions for each item [7, 17–19]. In Round 2 and subsequently, the experts were asked to rank the ‘appropriateness’ of each item using a 5-point scale (‘strongly disagree’, ‘disagree’, ‘undecided’, ‘agree’, ‘strongly agree’) and provide qualitative comments. In Round 2 or 3, the panelists could assess the views of other experts, allowing determination of the extent of group agreement (consensus if possible). Data were collected using an assessment form designed to capture responses to any specific problems noted and enable experts to refine their views as the rounds progressed. Consensus for a given item was considered achieved if response of ‘agree’ or ‘strongly agree’ was achieved by more than 75% of the expert panelists. The threshold for consensus was decided a priori. This threshold was deemed to reflect a general agreement among the substantial majority [18]. Based on previous Delphi studies, items that were rated as median ≥ 4 and by at least 75% of the panelists were included in the Norwegian language versions of the PFDI-20 and PFIQ-7 [7].

### Pilot test

The pilot test was modelled after the EORTC QoL Group translation patient face-to-face interview guide [13] and aimed to identify problematic items within the translated questionnaire from the perspective of the target group of the instrument (e.g., wording that caused confusion or words that were difficult to understand; to check equivalence and hesitations [3]). No hesitations in filling out the questionnaires indicated adequate linguistic validation or, as Guillemin et al. asserted, face validity [3].

## Outcomes

The application of the Delphi method in the Expert Panel phase proved adequate for producing a comprehensible Norwegian Intermediate Version 2.0 with few items of discrepancy and it showed semantic, conceptual, idiomatic, and experiential equivalence with the original versions. The Norwegian Intermediate Version 2.0 was then ready for pilot testing. The pilot testing undertaken during this study provided evidence that the Norwegian Intermediate Version 2.0 had a clear set of items with few discrepancies and no hesitations. After pilot testing, the Expert Panel reviewed comments from the patients, rendering the final translation Version 3.0 ready for testing of measurements properties.

Eleven minor discrepancies were identified during the pilot test and discussed in the final Expert Panel meeting, resulting in 9 amendments. These were mainly based on issues of semantic equivalence in Norwegian Bokmål and specific domain terminology (e.g., PFDI-20 question 3 “sensation of heaviness” was replaced with “a feeling of heaviness” due to patient feedback). All the panellists indicated that the amendments were necessary. However, some panellists felt that the changes improved the questionnaire items only marginally.

The Expert Panel review phase led to identification of several discussion topics and themes. During the Expert Panel phase, the panelists received information about their answers and the anonymous answers of the other panelists, as well as a statistical collective opinion (using medians) (Tables 1, 2, 3, 4). This iterative process and information gave the panelists the opportunity to re-evaluate their previous response to see if they wanted to reassess and change their rating. Furthermore, throughout the rounds, several alternatives were reviewed, and the task of iteration resulted in the expert panel becoming more focused on problem solving (Tables 1, 2, 3, 4).

**Table 1:** Two examples of the feedback obtained from the expert panel during Round 1

Round 1—expert panel review using the Delphi method PFDI-20 summary results from Round 1					
PFDI-20 items	Achieved equivalence^a^ between the source and Norwegian version in all the four areas Idiomatic Conceptual Semantic Experiential	Lack of equivalence^b^ between the source and Norwegian version in all the four areas Idiomatic Conceptual Semantic Experiential	Reason for disagreement If no, which area(s) of equivalence are not met? why?^b^	Number of suggestions for alternative wording^c^ Some panellists disagreed, however did not make any suggestions for alternative wording	Suggestion for alternative wording in Norwegian Can you suggest a change?^c^
Question 10	5 Specialists	3 Specialists	Idiomatic equivalence	2 Suggestions	Alternative 1: *Har du ofte avføringslekkasje når avføringen er løs eller flytende?* Alternative 2: *Har du vanligvis avføringslekkasje når avføringen er løs eller flytende?*
Question 20	5 Specialists	3 Specialists	Idiomatic equivalence	2 Suggestions	Alternative 1: *Kjenner du ofte smerte eller ubehag i nedre del av magen eller underlivet?* Alternative 2: *Har du ofte smerte eller ubehag i nedre del av magen eller underlivet?*

During round 1 the expert panellists were asked to assess the following questions ^a^Have all four equivalences been met? ^b^If “No”, which one(s) is/are not met and why? ^c^Can you suggest a change or alternative wording. The expert panel was comprised of eight pelvic floor specialists

No measure of consensus was employed in round 1. Voting and consensus commenced during rounds 2–4 (see Table 2)

**Table 2:** Two examples of the feedback obtained from the expert panel during Round 2

Round 2—expert panel review using the Delphi method PFDI-20 summary results from Round 2					
PFDI-20 Items	Single forward version	Alternatives from Round 1 *The alternatives from Round 1* *are voted on in Round 2*	Outcome^a,b^	Consensus ^b^,^c^ % (Median)	Comment
Question 10	Single Forward Version: *Har du vanligvis ufrivillig avføring hvis avføringen løs eller flytende?*	Alternative 1: *Har du ofte avføringslekkasje når avføringen er løs eller flytende?* Alternative 2: *Har du vanligvis avføringslekkasje når avføringen er løs eller flytende?*	**Alternative 1**	**A1 100% (5)**	No comments
Question 20	Single Forward Version: *Kjenner du vanligvis smerte eller ubehag i den nedre delen av magen eller underlivet*	Alternative 1: *Kjenner du ofte smerte eller ubehag i nedre del av magen eller underlivet?* Alternative 2: *Har du ofte smerte eller ubehag i nedre del av magen eller underlivet?*	Voted on however no consensus reached A1 87.5% A2 87.5% To be discussed and voted on in Round 4	No consensus Both A1 and A2 reached the same percentage 87.5% (4)	No comments

The content of questions and analysis of responses were re-circulated for clarification until a clear set of items that had cross-culture equivalence was identified for inclusion. ^a^The Question used during rounds 2–4: Have all the equivalences being met and do you believe the item should be selected in the final PFIQ-20 and PFIQ-7 questionnaires?—state the degree of agreement with specific domain terminology and four areas of equivalence using the Likert scale: 1 strongly agree—2 disagree—3 undecided—4 agree—5 strongly agree. ^**b**^Letters and Numbers in bold means that consensus has been reached with no further comments or rounds required. ^c^Consensus is defined as those items rated as median > 4 by at least 75% of the expert panellists with no additional comments

**Table 3:** Two examples of the feedback obtained from the expert panel during Round 3

Round 3—expert panel using the Delphi method PFDI-20 summary results from Round 3					
PFDI-20 Items	Round 2 version	New alternatives and suggestions made during Round 2 *These alternatives made in Round 2 are voted on in Round 3*	Outcome	Consensus^a^	Comments
Question 10			Consensus reached in Round 2		
Question 20	Alternative 1: *Kjenner du ofte smerte eller ubehag i nedre del av magen eller underlivet?* Alternative 2: *Har du ofte smerte eller ubehag i nedre del av magen eller underlivet?*	No new alternatives	Voted on however no consensus reached A1 87.5% A2 87.5% To be discussed and voted on in Round 4	No consensus Both alternative 1 and 2 reached the same percentage 87.5% (4)	No comments

^a^Consensus is defined as those items rated as median > 4 by at least 75% of the expert panellists with no additional comments

**Table 4:** Two examples of the feedback obtained from the expert panel during Round 4

Round 4—expert panel using the Delphi method PFDI-20 summary results from Round 4					
PFDI-20 Items	Round 3 Version ) *Few changes have made been made from Round 2*	New Alternatives and Suggestions made during Round 3 *These alternatives made in Round 3 are voted on in Round 4*	Outcome	Consensus^a,b^ (Median)	Comments
Question 10			Consensus reached in Round 2		
Question 20	Alternative 1: *Kjenner du ofte smerte eller ubehag i nedre del av magen eller underlivet?* *Alternative 2: Har du ofte smerte eller ubehag i nedre del av magen eller underlivet?*	No new alternatives	Alternative 2	A1 62.5% (4) **A2 100% (4)**	No comments

^a^Consensus is defined as those items rated as median > 4 by at least 75% of the expert panellists with no additional comments. ^b^Letters and numbers in bold means that consenus has been reached with no further comments or rounds required

Although the overall agreement was that the inclusion of qualitative data improved cross-cultural adaptation quality, panel members also agreed that the procedure was time-consuming. However, no panelist suggested that the Delphi method should not have been used. Panel members further noted the value of the opportunity for controlled feedback, which gave them time to assess and evaluate the group judgment. Finally, internal logic was evident because, for many items, panel agreement increased as the process evolved.

Aspects of professional asymmetry were evident during the Expert Panel review phase. During rounds 2 and 3, two panelists commented several times that their opinions were perhaps not as valuable. However, these panelists were among the most active members of the group, contributing several suggestions that were incorporated into the result. After the final meeting, one of the panelists expressed surprise that other members supported their proposal. These comments indicated that health professionals often feel a degree of professional asymmetry and different levels of hierarchy [20]. The translation co-ordinator also observed that during the Round 4 meeting two Expert Panel members dominated the group in the decision-making process. The dynamics of health professions are challenging, and the Delphi method in the Expert Panel situation can be beneficial when dealing with a dominant panelist. Anonymity was useful in this situation to avoid domination of the communication process by particular panel members based on their profession, age, or personality [6, 17]. This factor facilitated a situation in which all panelists felt that they could express their opinions freely and share their extensive field of knowledge.

Of interest, the Expert Panel almost unanimously voted for or against suggested phrasing of an item on many questions. Analysis of the PROM subscales containing these items also revealed that the panel was extremely efficient in evaluating the results of the initial translation stages for items involving clinical domain terminology. Notably, during the pilot test only 4/11 amendments were recommended due to specific domain terminology. This indicated that the target population understood the majority of the terminology used by the translators and the expert panel. To assist in this, the translators were briefed concerning the target population, culture, the content, and aim of the questionnaire [21]. However, some panelists pointed out that a layperson would seldom use Latin words to describe anatomical structures in Norwegian and that this could result in misunderstandings and ambiguities [15, 22]. For example, several panelists pointed out that the Norwegian layman term ‘skjeden’ was a better term than the Latin based ‘vagina’. The importance of a multidisciplinary Expert Panel was evident throughout the rounds. Each domain specialist contributed to the various subscales in the questionnaires.

## Discussion

Efforts to ensure a good translation and cross-cultural adaptation of the PFIQ-20 and PFIQ-7 from English to Norwegian led to the development of a new study methodology, using a Delphi approach with a bilingual pelvic floor Expert Panel. The method was effective in producing a Norwegian PFDI-20 and PFIQ-7 Intermediate Version 3.0 with a clear set of items that showed semantic, conceptual, idiomatic, and experiential equivalence with the original versions thus providing an adequate translation and cross-cultural adaptation.

Of note, incorporating controlled feedback into the Expert Panel in the form of a quantitative statistical representation provided a far more precise measure of the panel’s collective opinion and degree of consensus than having a face-to-face expert panel meeting with no formal voting systems. In addition, the Delphi method proved to be a highly structured, systematic communication technique with a rigorous documentation process. This systematic communication technique and documentation process can help elicit an even more rigorous procedure, which is often recommended by international translation task forces, within translation and cross-cultural adaptation. The modified Delphi method is a final face-to-face meeting that goes beyond the original Delphi method to address the unresolvable items of discrepancy.

To our knowledge, this study is the first to use novel translation methodology, including EORTC guidelines, Expert Panel review, and a Delphi approach, to achieve translation and cultural equivalence of such instruments. Significantly, the translation and cross-cultural adaptation of the PFIQ-20 and PFIQ-7 will provide improved assessment tools in this overlooked field of clinical practice in Norway. This iterative approach enabled the panel time to assess the group judgment, revise and improve ideas and by doing so, improve cross-cultural adaptation. Anonymity and statistical group response also improved the cross-cultural adaptation between rounds and ensured that input from every member of the panel was considered during the process and final response. Hence, anonymity, the iterative nature, and internal logic of the Delphi method seemed to improve cross-cultural adaptation because it identified and addressed limitations within the translation and cross-cultural adaptation method, namely back-translation and back-translation review. Task Force for Translation and Cultural Adaptation (ISPOR task force TCA) [23] and other authors acknowledge the importance of a back-translation review for cross-cultural adaptation [1, 13, 23]. However, Swaine-Verdier et al. [24] and other authors assert that back-translation is merely another way of checking, and clearly a scientific basis for back-translation is lacking [25–27]. This study also demonstrated the limitations of the back-translation and review phases. A situation arose in which the single forward translation seemed too literal and appeared too close to formal aspects of the original version in terms of syntax. The back-translations should have revealed this issue but instead indicated that the single forward translation was adequate.

Nevertheless, the expert panel through its iterative nature and internal logic of the Delphi method identified these shortcomings of the back-translation and more importantly, demonstrated a need for a more comprehensive multistep (i.e., Delphi consensus method with an expert panel, expert panel review and pilot testing after cross-cultural adaptation) for rechecking and identifying poor specific domain terminology, and semantic, idiomatic, conceptual, and experiential equivalence.

While several other translation and cross-cultural adaption methods exist [2] a gold standard has yet to be established [24]. With no gold standard, the Delphi consensus method with an expert panel, expert panel review and pilot testing can be used and applied to most translation and cross-cultural adaptation methods to help identify poor specific domain terminology and equivalence from orginal versions.

The Norwegian PFDI-20 and PFIQ-7 Intermediate Version 3.0 were ready for further extensive evaluation of measurement properties including reliability, validity, and responsiveness at baseline and after surgical treatment. Norwegian translations of the PFDI-20 and PFIQ-7 were demonstrated to have adequate reliability, content and construct validity, responsiveness to change, and interpretability [14]. However, cross-cultural validation was not performed on the Norwegian PFDI-20 and PFIQ-7 Intermediate Version 3.0. This type of validation process determines whether the items have the same meaning, compared to the original instrument, after the translation [25, 28].

Finally, more studies are evidently needed in this area of research to examine whether this method is suitable, viable, and reliable for translation andcross-cultural adaptation.

### Strengths and limitations

The strengths of this study included the use of a mixed methodology in the translation and cross-cultural adaptation of the PFDI-20 and PFIQ-7 to produce a data-rich evidence base (i.e., forward- and back-translations), reinforced with qualitative and quantitative evaluations (i.e., the Delphi consensus method with an expert panel).

Study limitations included that members of the Expert Panel considered the study to be time consuming. This perception could led to drop-out or at least loss of interest [16], with the consequence of ‘agreement’ without full evaluation. Second, especially when compared to other translation methods, it was difficult to assess and measure whether the Delphi method employed during the Expert Panel phase did, in fact, improve the quality of the cross-cultural adaptation. Third, on the feedback form completed by the Expert Panel, the response option ‘undecided’ could be interpreted as being unable to answer the question. The option ‘neutral’ might have reduced participant misunderstanding [1]. Last, the criteria changed between rounds, which could have created bias in the analysis of data. Round 1 was designed to collect options from the panelists and encourage them to suggest alternative wordings [17, 18]. Rounds 2, 3, and 4 were aimed at achieving consensus [17, 18] by voting using a 5-point scale. This scale could have been used in all rounds but carried the risk of not drawing several new suggestions for alternatives [17, 18].

A multistep procedure was important in improving equivalence and ensuring good cross-cultural adaptation during the translation of the PFDI-20 and PDIQ-7 [3]. This method ensured a rigorous cross-checking system during the whole process, particularly before and after back translation; back-translation review, and pilot testing. However, discrepant items may have been difficult to resolve without domain-level expertise. This difficulty was alleviated by using a multidisciplinary Expert Panel. Finally, the overall translation procedure might have been improved by giving the Expert Panel more information around the problematic items identified during the early steps of the process. However, in the current study, the consequence of withholding information on problem items yielded a verification effect that helped to confirm which persistently difficult items should be included in pilot testing.

### Future research

We recommend further evaluation of the applicability and viability of the multi-step method described. Several alternatives to the Delphi method with an Expert Panel exist, including the nominal group technique and multi-voting [29]. Future research could reasonably include comparisons with other such methods, with the aim of developing a gold standard process for translation, cross-cultural adaptation, and validation of HRQoL and similar measures. The proposed study would aid in further assessment of the iterative nature, and internal logic of the Delphi consensus method, in particular, the system of anonymity, in improving cross-cultural adaptation results.

Further, after translation of HRQoL measures using a Delphi method, cross-cultural validation would be recommended to ensure conceptual equivalence [25, 28]. That is, that the characteristics of the proposed instrument are comparable to those of the source instrument. Cross-cultural validation is an integral part of testing measurement properties and is normally performed using confirmatory factor analysis, differential item functioning analysis, or item response theory techniques [25, 28].

## Conclusion

This study presents a new methodology for translation and cross-cultural adaptation of two PROMs, the PFDI-20 and PFIQ-7, by using a Delphi method with a bilingual pelvic floor Expert Panel. To our knowledge, this study was the first to use this novel translation methodology. The thorough method resulted in a Norwegian PFDI-20 and PFIQ-7 Intermediate version 3.0 that was tested for measurement properties, and demonstrated adequate reliability, content and construct validity, responsiveness, and interpretability [14].

The rigorous documentation process, controlled feedback approach (in the form of a quantitative statistical representation), iterative nature, and internal logic of the Delphi consensus method appeared to ensure a good cross-cultural adaptation of these questionnaires. Finally, anonymity improved the cross-cultural adaptation between rounds and ensured that input from every member of the panel was considered throughout the process and in the final response. However, further studies are needed to determine whether this approach is a feasible and reliable translation method more generally.

## Data Availability

The datasets used and/or analysed during the current study are available from the corresponding author on reasonable request.

## Abbreviations

PROMs: Patient-reported outcome measurements
HR: Health-related
QoL: Quality of life
POP: Pelvic organ prolapse
PFDI-20: Pelvic Floor Distress Inventory
PFIQ-7: Pelvic Floor Impact Questionnaire
EORTC: European Organization for Research and Treatment of Cancer
ISPOR task force TCA: Task Force for Translation and Cultural Adaptation
